# Constant hydraulic supply enables optical monitoring of transpiration in a grass, a herb, and a conifer

**DOI:** 10.1093/jxb/erac241

**Published:** 2022-06-21

**Authors:** Ibrahim Bourbia, Christopher Lucani, Timothy J Brodribb

**Affiliations:** School of Natural Sciences, University of Tasmania, Hobart, Tas, Australia; School of Natural Sciences, University of Tasmania, Hobart, Tas, Australia; School of Natural Sciences, University of Tasmania, Hobart, Tas, Australia; Hong Kong Baptist University, Hong Kong

**Keywords:** Canopy transpiration monitoring, optical dendrometer, root to stem hydraulic conductance, water potential

## Abstract

Plant transpiration is an inevitable consequence of photosynthesis and has a huge impact on the terrestrial carbon and water cycle, yet accurate and continuous monitoring of its dynamics is still challenging. Under well-watered conditions, canopy transpiration (*E*_c_) could potentially be continuously calculated from stem water potential (Ψ_stem_), but only if the root to stem hydraulic conductance (*K*_r-s_) remains constant and plant capacitance is relatively small. We tested whether such an approach is viable by investigating whether *K*_r-s_ remains constant under a wide range of daytime transpiration rates in non-water-stressed plants. Optical dendrometers were used to continuously monitor tissue shrinkage, an accurate proxy of Ψ_stem_, while *E*_c_ was manipulated in three species with contrasting morphological, anatomical, and phylogenetic identities: *Tanacetum cinerariifolium*, *Zea mays*, and *Callitris rhomboidea*. In all species, we found *K*_r-s_ to remain constant across a wide range of *E*_c_, meaning that the dynamics of Ψ_stem_ could be used to monitor *E*_c_. This was evidenced by the close agreement between measured *E*_c_ and that predicted from optically measured Ψ_stem_. These results suggest that optical dendrometers enable both plant hydration and *E*_c_ to be monitored non-invasively and continuously in a range of woody and herbaceous species. This technique presents new opportunities to monitor transpiration under laboratory and field conditions in a diversity of woody, herbaceous, and grassy species.

## Teaser

The water potential and shrinkage of leaves are shown to be linearly related to transpiration rate in irrigated plants, providing the potential to monitor transpiration using optical methods.

## Introduction

Plant transpiration is an unavoidable consequence of biomass production and a key component of the terrestrial water cycle (Schlesinger and Jasechko, 2014). Maximum transpiration generally occurs in moist soil where stomata are not forced to close by leaf water deficit generated directly by drying soil, or by reductions in soil–leaf hydraulic conductivity (Scoffoni and Sack, 2017; Rodriguez-Dominguez and Brodribb, 2019; Carminati and Javaux, 2020; Abdalla *et al.*, 2021; Bourbia *et al.*, 2021). Quantifying the dynamic behaviour of transpiration under non-stressed conditions is necessary to explain the atmospheric cycling as well as temporal dynamics of carbon assimilation and plant water use under varying atmospheric conditions (Brodribb *et al.*, 2020). However, continuous *in situ* estimation of plant transpiration is still a challenging task due to technical limitations.

Few techniques have been developed to continuously quantify transpiration at the plant level. Sap flow measurement is perhaps the most commonly applied method, but this has limited temporal resolution and is typically restricted to measuring woody plants (Granier, 1987; Steppe *et al.*, 2015; Tfwala *et al.*, 2018). Gravimetric methods can provide precise information, but are really only suitable for small (potted) plants (Herbst *et al.*, 1996). Other methods relying on meteorological variables, such as the Bowen ratio energy balance (Spittlehouse and Black, 1980; Malek and Bingham, 1993) or eddy covariance systems (Baldocchi *et al.*, 2001; Williams *et al.*, 2004), and satellite-based remote sensing combined with complex modelling (Allen *et al.*, 2007; Senay *et al.*, 2013; Reyes-González *et al.*, 2018) have been also employed for larger scale measurements. However, these techniques are unable to partition soil and plant water loss and differentiate transpiration between species in spatially heterogenous environments (Tang *et al.*, 2006; Schlesinger and Jasechko, 2014; Bai *et al.*, 2019; Nelson *et al.*, 2020). Therefore, they are unsuitable for studying temporal dynamics of a species-specific water budget or competition for water between different species.

Canopy transpiration (*E*_c_) is strongly influenced by plant hydraulic conductance, mainly root–stem conductance (*K*_r-s_; here denoting the flow path from the root surface to the stem xylem) which is known to be the lowest within the liquid component of the soil–plant–atmosphere continuum and the most sensitive to water stress (Brodribb and Cochard, 2009; Rodriguez-Dominguez and Brodribb, 2019; Bourbia *et al*., 2020, 2021). The relationship between *K*_r-s_ and *E*_c_ can be described using Darcy’s law in the form *E*_c_= *K*_r-s_×ΔΨ (Equation 1) (Sperry *et al.*, 1998), where ΔΨ is the water potential difference between the root surface (Ψ_soil_) and the stem xylem (Ψ_stem_). Based on Equation 1, if *K*_r-s_ remains constant when *E*_c_ changes, clearly ΔΨ should be directly proportional to the *E*_c_ at equilibrium. In this case ΔΨ could be used as a sensitive proxy for *E*_c_. Clearly this approach would not be valid under conditions of drought because *K*_r-s_ has been shown to decline drastically during the early stages of water stress (around –1 MPa; Ψ_soil_), triggering stomatal closure in different species (Blizzard and Boyer, 1980; North and Nobel, 1991; Cuneo *et al*., 2016, 2021; Rodriguez-Dominguez and Brodribb, 2019; Bourbia *et al.*, 2021). However, under relatively wet soils (0≥ Ψ_soil_≥ –1 MPa), where most transpiration typically occurs, *K*_r-s_ may be sufficiently stable to allow monitoring by ΔΨ proxy. Yet this assumption of constant *K*_r-s_ in unstressed plants is still uncertain due to various reports that root *K* changes in response to *E*_c_ (Boyer, 1985). Some studies argue that, in well-watered plants, ΔΨ remains constant over a wide range of *E*_*c*_ rates, suggesting that *K*_r-s_ is dynamic (i.e. *K*_r-s_ increases and decreases with diurnally changing *E*_c_) (Macklon and Weatherley, 1965; Tinklin and Weatherley, 1966; Aston and Lawlor, 1979; Black, 1979). A similar number of papers present data indicating, on the contrary, that ΔΨ varies in a linear manner with *E*_c_ in other species, indicating constant *K*_r-s_ with changing *E*_c_ (Hailey *et al.*, 1973; Neumann *et al.*, 1974; Dubé *et al.*, 1975; Bunce, 1978; Ike *et al.*, 1978; Hirasawa and Ishihara, 1991).

In species displaying constant *K*_r-s_ under non-limiting water conditions where Ψ_soil_ was close to zero, Ψ_stem_ could be used to monitor *E*_c_, potentially providing a new window into the dynamics of plant water use. However, the main hurdle for employing such an approach is to accurately resolve the dynamics of Ψ_stem_ under variable field conditions. Psychrometers are the most widely used instruments for continuous monitoring of the *in situ* Ψ_stem_. However, their application is limited by installation difficulties, particularly on soft herbaceous plants, and the low degree of stability under fluctuating temperatures (Dainese *et al.*, 2022). Alternatively, several studies have shown that plant Ψ_stem_ can be readily estimated, indirectly but accurately, using tissue width variation (e.g. petioles and branchlets) because of the strong linear relationship found between these two parameters across a wide range of Ψ_stem_ (Bourbia *et al.*, 2021). Stem width variation can be recorded continuously using automated dendrometers (Klepper *et al.*, 1971; Fereres and Goldhamer, 2003; De Swaef *et al*., 2009, 2015), but a newly described optical dendrometer provides sufficient resolution to measure changes in tissue width in determinate (non-growing) structures such that changes in petiole or leaf width over extended periods can be used to monitor plant water status (Bourbia *et al.*, 2021). Optical dendrometers thus have the potential to continuously track daily fluctuations in plant transpiration at high temporal resolution using tissue width changes as a proxy for transpiration.

With the aim of assessing the potential for *E*_c_ to be monitored optically using dendrometers attached to leaves, we first investigated whether *K*_r-s_ is constant or a function of changing *E*_*c*_ in well-watered individuals of three divergent species. We use three phylogenetically, morphologically, and ecologically divergent species—*Tanacetum cinerariifolium* is a perennial herb with herbaceous roots, *Zea mays* is a grassy monocot which also has herbaceous roots, and *Callitris rhomboidea* is a hardy conifer with woody roots—in an attempt to test the generality of our findings. Establishing that *K*_r-s_ remains static in each species, we test the accuracy of predicting dynamic *E*_c_ from variation in petiole or leaf shrinkage.

## Materials and methods

### Plant material and growth conditions

Plants of *Tanacetum cinerariifolium* (Trevir.) Sch. Bip, *Zea mays* L, and *Callitris rhomboidea* R. Br. ex A. Rich. & Rich. were grown from seeds or rootstock (in the case of *T. cinerariifolium*) in glasshouse facilities at the University of Tasmania. Due to the different growth characteristics of the species, plants were potted in the optimum growth medium which was different for the different species. *Tanacetum cinerariifolium* plants were grown in 5 litre pots containing natural soil (clay) obtained from northwest Tasmania where it is grown commercially. The woody *C. rhomboidea* seedlings ranged from 30 cm to 40 cm in height and were potted in 2 litre pots using potting mix (medium 7:4 mix of composted fine pine bark and coarse washed river sand). *Zea mays* plants were gown in 2 litre pots filled with loamy soil. All plants were grown under unfiltered natural light in a controlled glasshouse cell at 25/15 °C day/night temperature and 40/80% day/night relative humidity (RH), and were watered daily to field capacity. Plants of *Z. mays*, *T. cinerariifolium*, and *C. rhomboidea* used in this experiment were ~2, 6, and 14 months old, respectively.

### *K*_r-s_ response to changes in *E*_c_

*K*_r-s_ was determined at high and low rates of steady-state whole plant transpiration (*E*_c_, mmol m^−2^ s^−1^) to verify whether it is static or dependent on *E*_c_ or Ψ_stem_. *K*_r-s_ was calculated based on the normal application of Darcy’s law standardized to viscosity of water at 20 °C:


$$\begin{array}{l} K_{r-s}\;=\;\frac{Ec}{{\;\Psi \;}_{soil}-{\;\Psi \;}_{stem}\;}\; \end{array}$$ (2)

This was done by simultaneously and continuously measuring *E*_c_ and Ψ_stem_ in well-watered plants subjected to different transpirational demands by manipulating RH in a controlled chamber as described below. Ψ_soil_ was assumed to be 0 MPa because pots were watered before and throughout measurements. Steady-state conditions refer here to conditions where both *E*_c_ and corresponding Ψ_stem_ are at steady state under stable RH, meaning that the plant capacitance effect is negligible.

### Continuous measurements of *E*_c_ and Ψ_stem_

On the evening preceding measurements, pots of four plants per species were watered and allowed to drain excess water then covered with a plastic bag to prevent evaporation from the soil. Plants were then transferred to a well-ventilated controlled-environment chamber, placed on computer-interfaced balances, and weighed continuously (every 5 min) to an accuracy of ±0.01 g (model PG5002-S; Mettler Toledo, Columbus, OH, USA). In each individual plant, a high-resolution automated optical dendrometer (Cavicam Co, Hobart, Australia) (for details see www.cavicam.co and Bourbia *et al.*, 2021) was attached to a mature (non-growing) petiole (*T. cinerariifolium*), leaf blade (*Z. mays*), and terminal branchlet (*C. rhomboidea*). The optical dendrometer was used to monitor width variation continuously (at 1–5 min intervals) from which the temporal dynamics of Ψ_stem_ could be inferred (see calibration details below) during *E*_c_ measurements.

**Fig. 1.**
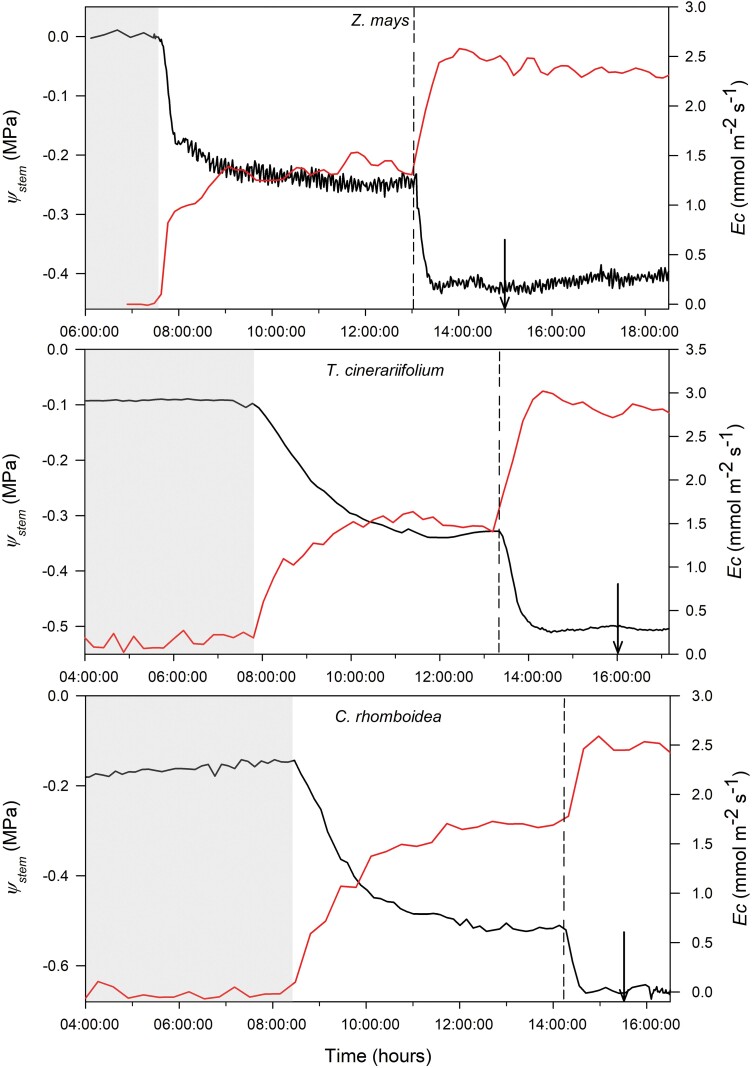
The response of stem water potential (Ψ_stem_; black line), inferred from foliar width variation measured continuously with optical dendrometers, to changes in whole-plant transpiration (*E*_c_; red line) measured gravimetrically in one representative individual of *Z. mays*, *T. cinerariifolium*m, and *C. rhomboidea* subjected to two RH levels under well-watered conditions. Temporal dynamics of Ψ_stem_ were monitored at 1 min intervals in *Z. mays* and at 5 min intervals in *T. cinerariifolium* and *C. rhomboidea*. The vertical dashed lines represent the time at which RH was decreased from 70% to 40–30%. Grey background, PPFD=0 μmol m^–2^ s^–1^; white background, PPFD=450 μmol m^–2^ s^–1^. Arrows indicate when pots were watered.

Prior to the transpiration treatments, plants were left in the dark during the night at 20 °C (for *T. cinerariifolium* and *C. rhomboidea*) and 25 *°*C (for *Z. mays*), and high RH (~90%). During the next morning, plants were illuminated at a photosynthetic photon flux density (PPFD) of 450 μmol quanta m^−2^ s^−1^ (at the canopy level) and RH was decreased to ~70% [vapour pressure deficit (VPD)=0.6 kPa for *T. cinerariifolium* and *C. rhomboidea*; VPD=1 kPa for *Z. mays*] and sustained using a commercial humidifier (SeccoUltra 00563, Olimpia-Splendid, Gualtieri, Italy) until *E*_c_ and leaf width reached stability and remained steady for at least 1–2 h, then RH was decreased to ~40–26% (VPD=1.6 kPa for *T. cinerariifolium* and *C. rhomboidea*; VPD=2.4 kPa for *Z. mays*) and maintained at this level using a dehumidifier until *E*_c_ and width reached a new steady state and remained stable for at least 1–2 h (Fig. 1). RH levels in the chamber were always modified in a regular sequence; from high RH in the morning to low RH at mid-day as described above. Temperature was held constant throughout measurement irrespective of RH change and was maintained at the same level as night temperature.

Foliar width was calibrated against Ψ_stem_ measured with a Scholander chamber (PMS, Albany, OR, USA) in each individual plant across a range of 2–3 Ψ_stem_ values encompassing the maximum range observed during transpiration treatments. These measurements were performed on non-transpiring mature leaves in *T. cinerariifolium*, branchlets in *C. rhomboidea*, and the tips of the uppermost fully expanded leaves in *Z. mays* (15 cm in length=one-third of the total leaf length) that were enclosed in a plastic bag covered with aluminium foil the night before measurements. One measurement was made in the dark before switching on the lights and the others were made at the two levels of RH to which the plants were exposed after width had reached a steady state.

Soil temperature in the centre of the pot was monitored using a thermocouple connected to a datalogger (CR850, Campbell Scientific, Logan, UT, USA). Soil temperature reached air temperature during the night, and both remained constant during measurements throughout the day (within 0.5 *°*C) independent of RH change.

Air temperature and RH in the growth chamber were monitored at 30 s interval with a temperature/humidity probe (HMP45AC; Vaisala Inc., Helsinki, Finland) placed close to the measured plants, and logged on the same datalogger.

During measurements, water lost by transpiration was added at the top of the pot periodically to avoid any drop in soil water potential (or soil hydraulic conductance) especially at high transpiration after decreasing RH. The water added into the soil was at the same temperature as the soil. *E*_c_ was normalized by the projected canopy leaf area measured at the end of the experiment with a flatbed scanner in all species.

### Predicting *E*_c_ from optically monitored Ψ_stem_ dynamics using a constant *K*_r-s_

According to Equation 2, if *K*_r-s_ remains constant, *E*_c_ can be estimated from Ψ_stem_ after correcting for viscosity. In each individual plant, we used plant-specific *K*_r-s_ values (corrected for changes in viscosity due to soil temperature) measured at high RH (and less negative Ψ_stem_) to estimate *E*_c_ in the same individual at low RH from Ψ_stem_ inferred from petiole/leaf width measured with the optical dendrometer. This predicted *E*_c_ was compared with *E*_c_ measured gravimetrically under these conditions. To determine the error associated with using species mean *K*_r-s_ rather than individual *K*_r-s_, mean measured *K*_r-s_ of each species was also used to predict *E*_c_ in individual plants of each species at varying measured Ψ_stem_ levels, and the values were compared with the measured *E*_c_ values.

### Statistical analysis

Differences between species mean values of *K*_r-s_ were tested with Student’s *t*-tests after testing for normality and homogeneity of variances. We used linear regressions to quantify the correlation between tissue width variation and Ψ_stem_ in each plant individual used for *K*_r-s_ measurements. Results are presented as mean values ±SE. Differences were considered to be significant when *P*<0.05. The accuracy of predicted *E*_c_ relative to the observed *E*_c_ was computed using the mean absolute percentage error metric: $MAEP=\frac{100}{n}\underset{i=1}{\overset{n}{\sum }}\;\left|\frac{Ai-Bi}{Ai}\right|,$ where *Ai* is the actual value, *Bi* is the predicted value, and *n* is the total number of observations. All analyses were performed using R version 3.5.3 (R Core Team, 2019). Figures were created using Sigmaplot version 12.5 (Systat Software Inc., San Jose, CA, USA).

## Results

Leaf width monitored continuously with optical dendrometers was highly linearly correlated with measured Ψ_stem_ in each individual plant (*r*^*2*^=0.99, *P*<0.001) (Supplementary Fig. S1), allowing the Ψ_stem_ dynamics to be predicted at high temporal resolution and *in situ* from leaf/petiole width variation. Changes in Ψ_stem_, inferred from leaf/petiole width changes, followed *E*_c_ changes closely in all species (Fig. 1). Based on the calibrated optical dendrometers, Ψ_stem_ was found to fall and reach a new steady state quickly (within 30 min) once *E*_c_ increased in all species. There was no lag between *E*_c_ and a resultant change in Ψ_stem_ under non-steady-state conditions either in the morning after turning on the lights or at mid-day after changing RH (i.e. both *E*_c_ and Ψ_stem_ reached steady state at the same time).

**Fig. 2.**
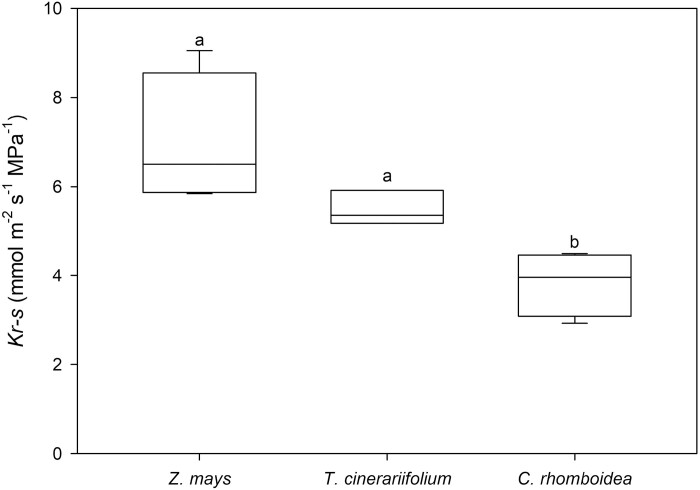
Mean measured root to stem hydraulic conductance (*K*_r-s_) in *Z. mays*, *T. cinerariifolium*, and *C. rhomboidea* under well-watered conditions (*n*=4±SE). Different letters indicate statistically significant differences at *P*<0.05.

Mean *K*_r-s_ varied considerably among species (*P*<0.05) and was significantly higher in the monocot *Z. mays* (6.94 ± 0.75 mmol m^−2^ s^−1^ MPa^−1^) and the herbaceous *T. cinerariifolium* (5.48 ± 0.16 mmol m^−2^ s^−1^ MPa) compared with the woody *C. rhomboidea* (3.84 ± 0.37 mmol m^−2^ s^−1^ MPa; Fig. 2). However, due to the variability in *K*_r-s_ among *Z. mays* plants (SD=22%), differences in mean *K*_r-s_ between this species and *T. cinerariifolium* were not significant. Significant variability in *K*_r-s_ was also observed between plants in *C. rhomboidea* (SD=19%) but it was very small in *T. cinerariifolium* (SD=6%).

### Constancy in *K*_r-s_

**Fig. 3.**
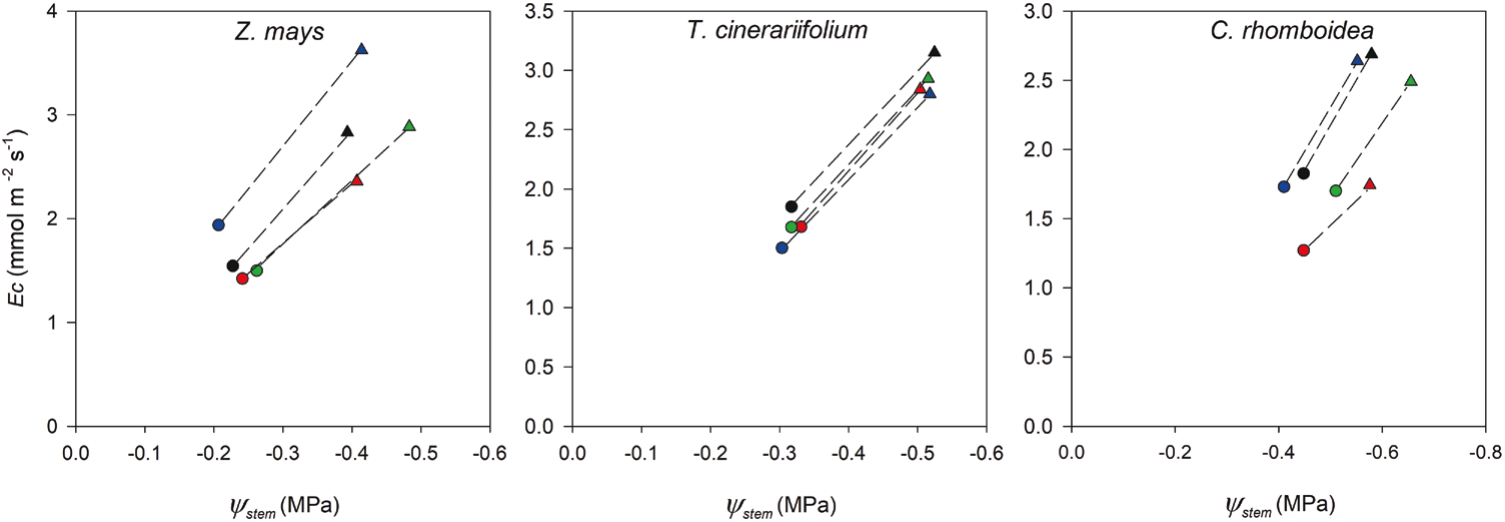
Response of stem water potential (Ψ_stem_) to changes in whole-plant transpiration (*E*_c_) achieved by varying RH in each individual plant of *Z. mays*, *T. cinerariifolium*, and *C. rhomboidea* under well-watered conditions. Both Ψ_stem_ and *E*_c_ presented here were in the steady state. Each colour represents an individual measured at high RH (circle) and low RH (triangle).

**Fig. 4.**
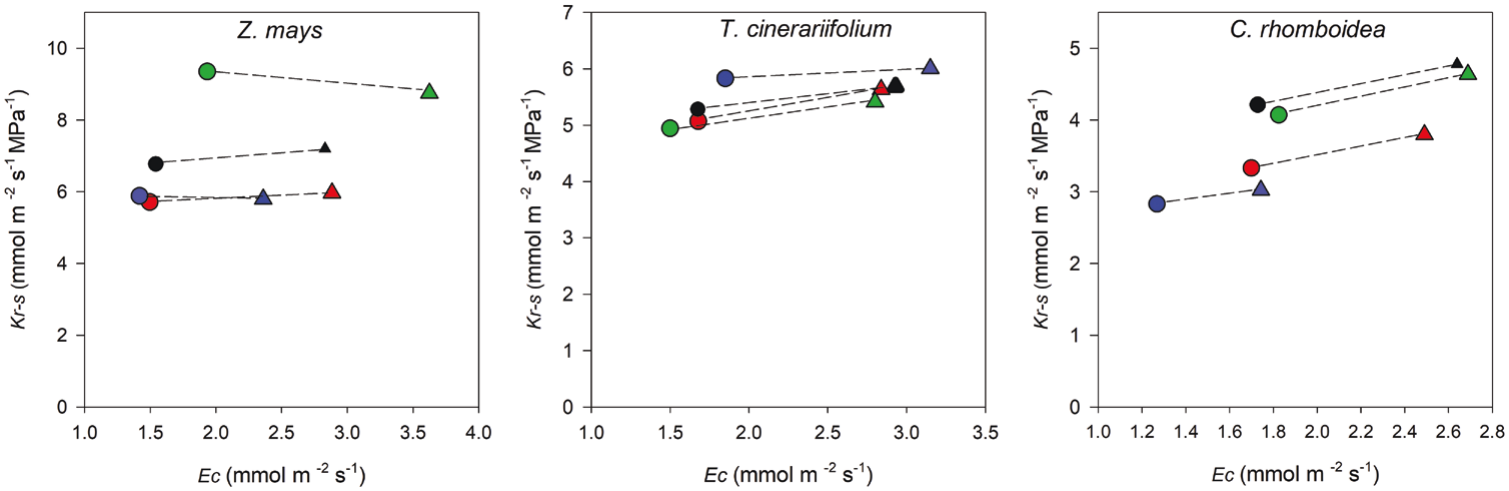
Response of root to stem hydraulic conductance (*K*_r-s_) to the increase in whole-plant transpiration (*E*_c_) induced by lowering RH in individual plants of *Z. mays*, *T. cinerariifolium*, and *C. rhomboidea* under well-watered conditions. Each colour represents an individual measured at high RH (circle) and low RH (triangle).

**Fig. 5.**
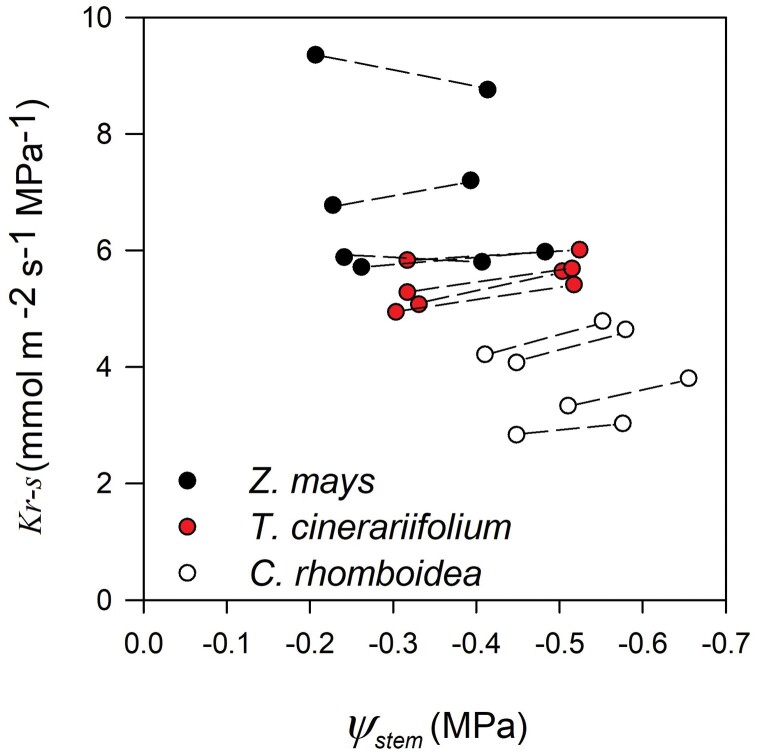
Response of root to stem hydraulic conductance (*K*_r-s_) to decreasing stem water potential (Ψ_stem_) induced by increasing whole-plant transpiration (*E*_c_) in each individual plant of *Z. mays*, *T. cinerariifolium*, and *C. rhomboidea* under well-watered conditions.

Across a range of RH designed to simulate typical daytime conditions, the steady-state *E*_c_ and Ψ_stem_ remained in constant proportion in each individual plant of the three species (Fig. 3), revealing a constant *K*_r-s_ with changing *E*_c_ in all species (Fig. 4). As a result, *K*_r-s_ also remained constant alongside changing Ψ_stem_ in all species (Fig. 5).

### Predicted versus observed *E*_c_

**Fig. 6.**
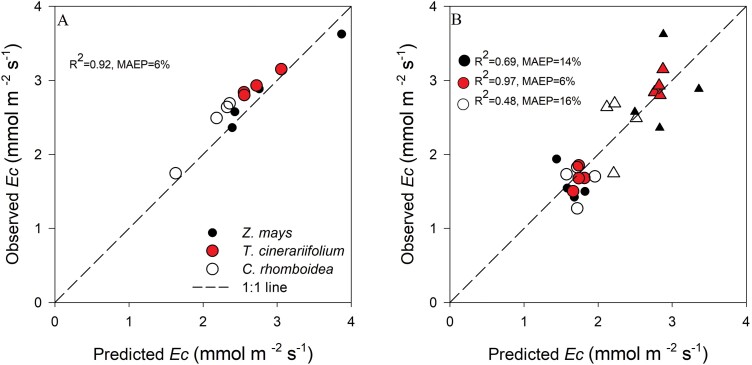
(A) Observed whole-plant transpiration (*E*_c_) measured at low RH compared with predicted values determined using individual *K*_r-s_ calculated at high RH. (B) Observed whole-plant transpiration (*E*_c_) compared with predicted values at low and high RH calculated using mean species *K*_r-s_. Circles and triangles in (B) refer to values *E*_c_ predicted at high and low RH, respectively.

According to Equation 2 applied during steady-state, the constancy of *K*_r-s_ should enable *E*_c_ to be predicted from monitored values of Ψ_stem_ (as measured by the optical dendrometer). In each individual plant, the predicted values for *E*_c_ at low RH calculated from individual *K*_r-s_ measured at high RH closely followed a 1:1 line (*R*^2^=0.92) and were within 5% (MAEP) of the measured values (Fig. 6A).

High predictive accuracy in *E*_c_ was also achieved when the species average *K*_r-s_ was used to estimate *E*_c_ at the individual level in the herbaceous *T. cinerariifolium* (*R*^2^=0.97, MAEP=6%) (Fig. 6B). However, this accuracy was limited by the substantial variation in the species mean *K*_r-s_ measured in *Z. mays* (*R*^2^=0.69, MAEP=14%) and *C. rhomboidea* (*R*^2^=0.48, MAEP=16%)

## Discussion

### *K*_r-s_ remains constant with changing *E*_c_

Our results demonstrated conclusively that, under steady-state conditions in hydrated soil, Ψ_stem_ varies in a proportional manner with *E*_c_, indicating that *K*_r-s_ remains constant with changing *E*_c_ in the three distinctly different species studied here (Figs 3, 4). Our results are consistent with those observed in several other species such as cotton, pea, cassava, rice, and sunflower (Hailey *et al.*, 1973; Ike *et al.*, 1978; Passioura, 1980; McBurney and Costigan, 1982; Hirasawa and Ishihara, 1991), and extend those presented by Neumann *et al.* (1974) and Dubé *et al.* (1975) for *Z. mays*. The consistent lack of variation in *K*_r-s_ in response to changes in *E*_c_ observed across our experimental species suggests that this is a consistent pattern across seed plants. Nevertheless, some studies reported *K*_r-s_ to vary with *E*_c_ in species grown in hydroponics (Macklon and Weatherley, 1965; Tinklin and Weatherley, 1966; Aston and Lawlor, 1979; Black, 1979). Macklon and Weatherley (1965) suggested that the decrease in Ψ_stem_ with increasing *E*_c_ usually observed in soil-rooted plants under field conditions is due to the steady drop in the hydraulic conductance of the rhizosphere (at the soil–root interface), occurring when water is absorbed more quickly at the root surface than it is replaced by that moving from untapped soils, even in well-watered conditions. They also argued that this does not occur in hydroponically grown plants because roots are constantly surrounded with water. Local dehydration around the root would violate the assumption in Equation 2 that soil Ψ_soil_=0 MPa. Here we avoided this possible error in *K*_r-s_ calculation by carefully adding water to the pot to ensure the water content remained saturated throughout the experiment. Furthermore, we did not observe any change in steady-state *E*_c_ or Ψ_stem_ after the addition of water at high transpirational demands (i.e. at RH=30%), suggesting that rhizosphere water potential remained close to zero (Fig. 1) and that our calculation of constant *K*_r-s_ was correct. Water flows radially across the root cylinder (from the root surface to the stele/xylem) along two parallel apoplastic and symplastic pathways, the latter being mediated by aquaporins. The discrepancy between our results and those discussed above regarding the constancy of *K*_r-s_ with changing *E*_c_ may be attributed to differences in the relative contribution of the apoplastic and cell to cell pathways to the whole *K*_r-s_ between different species during transpiration due to differences in root morphological and anatomical features (Kim *et al.*, 2018). A cell to cell transport of water has been shown to dominate in roots of some species such as barely (Steudle and Jeschke, 1983; Knipfer and Fricke, 2010) and bean (Steudle and Brinckmann, 1989), but to play a minor role in roots of maize (Steudle *et al.*, 1987), cotton (Radin and Matthews, 1989), and grapevine (Gambetta *et al.*, 2013) (i.e. water flows mostly through the apoplastic pathway). If water flows predominantly through the cell to cell pathway during transpiration, then *K*_r-s_ may change with *E*_c_ due to changes in aquaporin expression or activity (Maurel, 1997; Javot and Maurel, 2002; Knipfer and Fricke, 2011; Laur and Hacke, 2013). In the three species measured here, the constancy of *K*_r-s_ with changing *E*_c_ suggests that water flow in the roots follows a largely apoplastic pathway during transpiration under natural conditions.

In this study, *K*_r-s_ measured using the gravimetric method remained constant alongside decreasing Ψ_stem_ induced by increasing *E*_c_ in all species. This behaviour has also been demonstrated by Bourbia *et al.* (2021) in roots of *T. cinerariifolium* and *C. rhomboidea* measured with a non-steady-state rehydration technique over a wide range of Ψ_soil_ induced by drought (between –0.35 MPa and –1 MPa). This observation contrasts with that of a recent study in cotton (Wang *et al.*, 2020) which reported *K*_r-s_ to decline by >50% as Ψ_stem_ fell marginally from 0 MPa to –0.16 MPa. This surprisingly high sensitivity of *K*_r-s_ to water potential was measured on small segments of roots using the centrifuge technique which is known to produce contrasting results in roots depending on the pressure gradient protocols used (Bouche *et al.*, 2015). Our data support the conclusions of Bouche *et al.* (2015) that very high sensitivity of *K*_r-s_ to water deficit seen in centrifuge studies may be artefactual. However, it remains a possibility that a high sensitivity of *K*_r-s_ to water stress observed in cotton compared with other species could also be attributed to physiological and morphological differences between these species. Testing *K*_r-s_ in intact cotton plants would be a valuable step to confirm these results.

### Constancy of *K*_r-s_ allows *in situ* estimation of *E*_c_ from optically measured Ψ_stem_

The constancy of *K*_r-s_ observed here indicates that the dynamics of *E*_c_ can be calculated from Ψ_stem_ when it is at steady state according to Equation 2. The validity of using Ψ_stem_ as a proxy for *E*_c_ was evidenced by the strong agreement between gravimetrically measured *E*_c_ and that calculated from steady-state Ψ_stem_ at varying transpirational demands in all species (Fig. 6). The ability to continuously monitor Ψ_stem_*in situ* using optical dendrometers, as demonstrated in this study (Fig. 1), provides a promising approach for tracking *E*_c_ changes on a fine temporal scale in both woody and soft herbaceous species.

Unlike other plant-based methods, such as sap flux methods, optical dendrometers are very simple and easy to install, insensitive to external temperature variation, and very responsive to rapid changes in Ψ_stem_ (Fig. 1). Compared with microclimatological techniques (Bowen ratio and eddy covariance) which provide estimates of evapotranspiration, incorporating both plant and soil water loss (Williams *et al.*, 2004; Tang *et al.*, 2006; Schlesinger and Jasechko, 2014; Perez-Priego *et al.*, 2018), the optical technique measures plant transpiration alone, thus making it suitable for studying spatial and temporal dynamics of species-specific water use and carbon assimilation in mixed stands, and the responses to changes in climate in both natural and agricultural systems. This method can also be used, if scaled up to stand or regional level, as an independent ground-based method to validate models partitioning evaporation and vegetation transpiration (Lawrence *et al.*, 2007; Sutanto *et al.*, 2012), and as a tool to quantify irrigation demands in agricultural systems.

### Optical estimation of *E*_c_ under non-steady state conditions

The highly accurate estimations of *E*_c_ from optically derived Ψ_stem_ observed in the studied species were restricted to periods of steady-state conditions with no influence of plant capacitance (internal stored water). However, under field conditions, *E*_c_ may fluctuate substantially and rapidly over the course of the day in response to variations in climatic conditions, and seldom reaches a steady state (Jones *et al.*, 1982). In this case, the use of a steady-state model to predict instantaneous and fast changes of *E*_c_ from optically measured Ψ_stem_ can be valid only if the plant capacitance is negligible or its contribution to *E*_c_ is accounted for.

### Conclusion

The constancy of *K*_r-s_ under varying transpirational demands observed in this study was a common feature among different species. This means that the optical technique presented here can probably be used to estimate *E*_c_ in real-time in diverse plant species under steady-state conditions. Yet, further work is necessary to elucidate the applicability of this technique in monitoring instantaneous non-steady changes of transpiration under non-steady atmospheric conditions over the long term.

## Supplementary data

The following supplementary data are available at *JXB* online.

Fig. S1. Relationship between foliar tissue width and Ψ_stem_.

erac241_suppl_supplementary_figure_S1

## Data Availability

The data supporting the findings of this study are available from the corresponding author, Timothy Brodribb, upon request.
