# Supplementary figures and images for: Constant hydraulic supply enables optical monitoring of transpiration in a grass, a herb, and a conifer
Source: J Exp Bot. 2022 Jun 21;73(16):5625–33. doi: 10.1093/jxb/erac241 (PMC9467656; doi:10.1093/jxb/erac241)

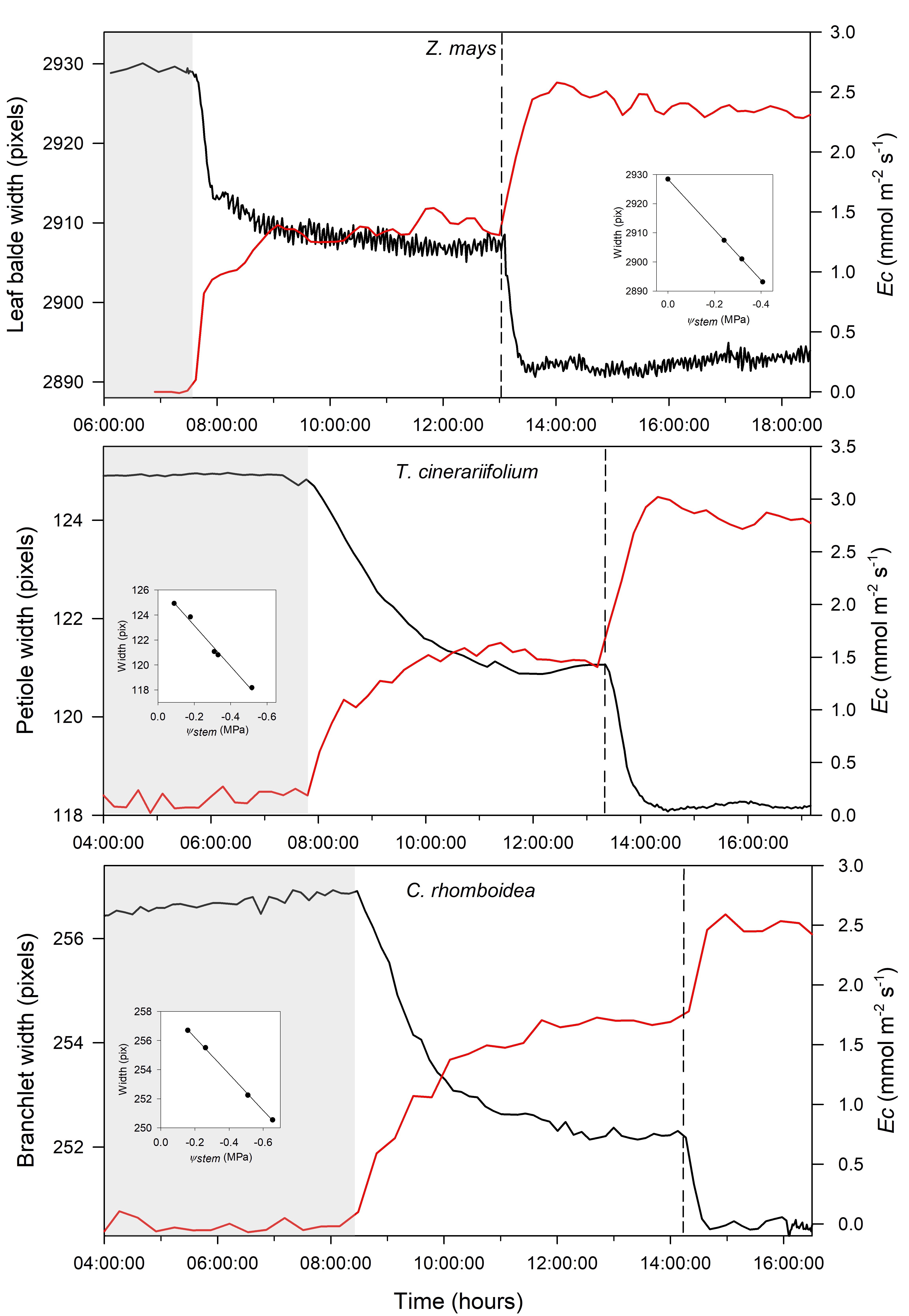

Supplement: erac241_suppl_supplementary_figure_S1 [file erac241_suppl_supplementary_figure_s1.jpeg]
